# An enigma of Malaysia’s low-income young adults: Mediation of financial behaviour on financial well-being and locus of control cohesion

**DOI:** 10.1371/journal.pone.0288204

**Published:** 2023-07-13

**Authors:** Mohamad Fazli Sabri, Rozita Wahab, Nurul Shahnaz Mahdzan, Amirah Shazana Magli, Husniyah Abd Rahim, Siti Shazwani Ahmad Suhaimi, Nur Shuhamin Nazuri

**Affiliations:** 1 Faculty of Human Ecology, Universiti Putra Malaysia, Serdang, Malaysia; 2 Malaysia Universiti Putra Malaysia, Serdang, Malaysia; 3 Faculty of Business and Economics, Department of Finance, University of Malaya, Kuala Lumpur, Malaysia; Universiti Teknologi Malaysia - Main Campus Skudai: Universiti Teknologi Malaysia, MALAYSIA

## Abstract

Young adults face many significant challenges to their financial well-being. The rising cost of living and unstable economies have impacted how they consume, manage, and save monthly income to maintain their standard of living. Hence, exploring the financial well-being of young adults in Malaysia is an intriguing and relevant research topic that deserves examination from multiple perspectives. This study aims to investigate how these three factors, namely financial knowledge and locus of control with financial behaviour as a mediator, are correlated with the financial well-being of low-income young adults in Malaysia. A total of 520 young adults from North, Central, South, East zones in Peninsular Malaysia and East Malaysia were randomly chosen using a multi-stage sampling technique as the sample of this study. Data in this study were obtained using a set of questionnaire-based survey through cross-sectional study and then scrutinized using IBM SPSS (Statistical Package of Social Science). This study discovered that financial knowledge, internal and external locus of control, and financial behaviour were significantly correlated with the financial well-being of low-income young adults. The findings also demonstrate that financial behaviour mediates the correlation between financial knowledge, both internal and external locus of control, and financial well-being. This study is one of the very few important studies that explore the link between financial literacy, locus of control, financial behaviour, and financial well-being among low-income young adults. This study also found an interesting and noteworthy fact regarding the impact of the minimum monthly wage policy on highly educated young adults in Malaysia, which is worth discussing and needs to be alerted to the policymakers and leaders of the country. Therefore, the findings of this study can be utilized as a starting point by policymakers, government organizations, and non-governmental organizations to create new initiatives aimed at raising financial well-being among the younger generation.

## Introduction

The issue of financial well-being (FWB) is crucial among young adults. In their daily routines, young adults are enticed to spend more money in their daily routines due to the trends of consumerism. Furthermore, due to the existence of e-commerce platforms, the purchasing process becomes easier than ever. People can also access financial assistance in a straightforward and hassle-free manner because of the ease of obtaining credit. Having a credit card is a necessity for most young adults regardless of their income level. Unfortunately, some of them struggle to financially survive, while relying solely on their monthly pay checks without credit cards [1].

In general, young adults aged 18 to 35 years [2, 3] comprise of several groups. These young adults could be newlyweds or new parents who are starting their own families and are responsible in taking care of their young children. They might also be jobless, recent graduates who are still looking for employment, or new hires at organizations with the lowest starting salaries compared to other senior staff. Young adults might also be students or student workers who are still in college and are in charge of their own daily expenses and tuition fees. This is a crucial phase in life when young adults need to start learning how to handle their money responsibly and make most of their own financial decisions, in contrast with adolescents, who are still receiving complete financial assistance from their guardians or older adults, who are more financially secure [4]. Young adults are at a point in life where they can benefit greatly from extra funds, yet they have a very limited or no assets and are not receiving much financial help from parents or relatives.

Findings from a survey done in United States reveal that 50% of young adults have nothing to put aside for rainy days [5]. With regard to their financial behaviour, the findings also showed that 32% of them can only afford to make the minimum payment of their credit card bills every month. Furthermore, a large percentage of young people (approximately 63%) fail to correctly respond to more than three of the five questions on a financial literacy test, indicating that many young adults lack basic financial knowledge, which in turn affects their ability to make wise financial judgements [6, 7]. It is also interesting to note that a growing percentage of Malaysian young adults are struggling to make their ends meet [8]. In addition, the number of young adults facing bankruptcy in Malaysia has increased over the years [9]. According to a report, 18,836 individuals under the age of 35 filed for bankruptcy between 2016 and 2020, accounting for 25.3% of all bankruptcy cases in Malaysia [10]. Thus, the financial issues surrounding young adults who are about to become financially independent is very worrying.

Meanwhile, it was previously discovered that approximately half of young employees between the ages of 18 and 35 were in serious debt [11]. The study also revealed that individuals earning RM 2,000 and RM 3,000 per month had the most severe debt problems [12]. In 2018, the Kredit Counselling and Debt Management Agency (AKPK) also conducted a survey which found that around half of all working adults in Malaysia had FWB scores in the ’require attention’ or ’under pressure’ range [13]. Two out of ten working adults claimed that they did not have any savings in the six months prior to the survey. To reiterate, more than half of the people whose annual income is less than RM20,000 do not even have at least RM1,000 for their emergency saving. Three out of ten also acknowledged that they occasionally had to borrow money to pay for basic needs. As a result, young adults heavily use credit cards and loans. These problems were plausibly caused by the purchase of assets such as houses and cars, which raised their debt repayment commitments. Considering these data, it comes as no surprise that young adults have reported lower FWB due to their poor financial behaviour and lack of financial knowledge.

As a first step toward being financially self-sufficient, young individuals should educate themselves with necessary knowledge on how to utilize credit cards wisely and best manage their savings account. [14, 15] asserted that in the early stages of adulthood, numerous important financial choices and decisions are made for the first time. As a result, young adults in this era seem to have had more trouble making personal financial decisions than previous generations [16]. This is partly because of the complicated consumer options they must deal with in the marketplace. Additionally, they are growing in an environment of uncertain and shifting economic conditions brought on by the collapse of financial markets, which has resulted in widespread financial hardship. Financial behaviour and psychological characteristics, such as locus of control (LOC) that are acquired throughout their lives have extensive ramifications since they persist until adulthood and lay the foundation for long-term FWB [17].

As previously defined, FWB is a positive circumstance with both objective and subjective aspects [18]. In this context, "entry" means a person’s income and "exit" means their debt; "resources" means both of these things and anything else the person has already obtained (e.g., education, property, savings accounts). Conversely, the subjective aspect refers to the individual’s psychological reaction and mental judgement of their financial circumstances. Most young adults begin the process of shifting from being financially dependent on their parents immediately after high school. Therefore, social scientists consider gaining financial literacy to be an important developmental milestone. What they learn and do during these formative years, both good and bad, will likely shape their choices for the rest of their lives [19]. The ability of locus of control (LOC) to accurately predict various life outcome variables, including FWB, has been evident in previous studies [20, 21]. LOC also affects behaviour and attitudes toward monetary matters [21]. Internal LOC is associated with a higher drive to achieve goals and a willingness to take responsibility for one’s actions, even financial ones [22].

Currently, young adults face a wide variety of significant obstacles to achieve high levels of FWB. Due to the consistently high cost of living, young adults in Malaysia are acutely aware of the challenges they face in terms of their financial stability. Considering these issues, they have had to adjust the way they purchase, invest, and save money, as well as how they deal with difficult situations, in order to keep their standard of living stable over the long term [23]. Despite the surge in research interest in FWB, it is still crucial for young adults because it can affect their physical, psychological, and social health, which can result in subpar job performance, lack of focus, decreased productivity, and disengagement from everyday activities [22]. As young people frequently find themselves in difficult financial situations and must make difficult financial decisions on their own, investigating the FWB of young adults in Malaysia is an intriguing and pertinent research issue that deserves to be investigated from a variety of viewpoints [4, 24].

Young people experience many difficulties in life and develop a wide range of life skills as they make a journey into adulthood. Among the most important life skills attained are those related to financial subjects. Previous studies have argued that financial matters, including financial well-being or financial satisfaction of an individual, are influenced by a variety of contextual factors, such as social norms, cultural values, and economic conditions. However, these factors are often overlooked in financial well-being studies [25, 26]. Past studies have paid limited attention to the social dimensions of financial well-being. Most financial well-being studies focus on individual-level factors, such as income, debt, and financial literacy, while overlooking the social dimensions of financial well-being, such as social class, in shaping financial well-being [26]. Many financial well-being studies also focus on relatively homogenous samples such as college students or middle-class households, which limits the generalizability of the findings to other populations [27]. Thus, studies focusing on the financial aspects of young adults are worthwhile, particularly among low-income groups. From the literature, it was found that there are studies focusing on financial aspects of vulnerable groups of society particularly the low-income households [28, 29]. However, there appears to be a very scant number of studies that address a specific segment of the vulnerable group that is the young adults of low-income households. Thus, these are the gaps that the present study aims to fill.

In light of this, the primary purpose of this study is to evaluate the execution of financial behaviour mediation on the association of FWB, financial knowledge, internal and external LOC amongst young adults in Malaysia, particularly among low-income groups. The idea of investigating financial behaviour as a mediator between financial knowledge and financial literacy as predictors with financial satisfaction or FWB as the outcome variable is based on previous studies [30–32]. The justification for the indirect relationship between financial knowledge and FWB is that FWB may not necessarily result from financial knowledge alone. Instead, the knowledge needs to be converted and manifested into positive financial behavior to obtain the desired outcomes, that is, high FWB.

This study applied the System Theory as a main theory to explain the interaction between the input and the Malaysian low-income young adult’s financial well-being (output). According to Ludwig von Bertalanffy’s System Theory, which was first proposed in 1968 and has since been refined, there are two components to the world: the environment and the system [33]. Interactions between a system and its environment are evaluated by comparing the system’s outputs to its outcomes or objectives. The Family Resource Management Model, also serves as a foundation for this research as it was developed from the System Theory [34]. The model has garnered a lot of attention and support from the personal finance literature that came before it [35, 36]. The three primary components of this model, namely input, throughput, and output, were described in order to provide an explanation of how a family makes plans and utilizes resources in order to satisfy the requirements of the consumption process. As according to [32, 37], understanding financial decision-making and the consequences of those decisions requires consideration of these three components. Input refers to resources, such as individual skills and knowledge. Throughput refers to the process of planning and taking action to satisfy goals. Lastly, outputs refer to the outcomes of planning and action taken. It gives people an entire impression of whether or not the goal has been achieved [34]. In the present study, financial knowledge, internal LOC and external LOC as input while throughput and output are represented by financial behaviour and financial well-being respectively. The input-throughput-output interaction offers a theoretical ground for this study to generate the desired result, which in this case is good financial well-being. Low-income young adults can benefit from better financial well-being by enhancing their savings habits (financial behaviour), financial knowledge, including both internal and external LOC.

This study contributes to the existing body of knowledge by adopting financial behaviour as a mediator between financial knowledge, LOC, and FWB, which is a relatively unexplored area in the existing literature. Previous research has typically focused on the direct connections between LOC and financial behaviour [20, 38–40], the direct connections between LOC and FWB, satisfaction, or wellness [22], and the mediating effect of LOC on the associations between certain antecedents and other financial consequences, including FWB [22, 41–43]. However, in the present study, researchers use financial behaviour as a mediator in the relationship between two dimensions of LOC (internal and external), with FWB. The rationale for adopting financial behaviour as a mediator is similar to the earlier argument, in which the researchers suggest that LOC (both internal and external) will not by itself have an impact on FWB, but instead needs a pathway that will enable the psychological trait to be converted into favourable financial outcomes. The researchers argue that individuals with either high internal LOC or low external LOC will be able to demonstrate and engage in prudent financial behaviour that will allow one to realize high levels of FWB. This study differs from past studies that have measured LOC as a single construct rather than to differentiate between internal and external LOC. For example, [19, 20] who examined the mediating effect of financial behaviour on the relationship between LOC and FWB, combined both dimensions of LOC into one and did not differentiate between the two dimensions. Separating LOC into the two dimensions (internal and external LOC) may prove to provide more insights on whether it is the belief of one’s own actions that can influence the outcomes in a person’s life, or do external factors such as luck, the environment or other people have more influence on life’s satisfaction and well-being. If the findings reveal that external LOC is a significant factor contributing towards low FWB, then this has important implications for policy makers to develop policies that could empower these young adults to take charge of their lives by providing the necessary skills and training that could assist them to help themselves escape from the poverty trap. In Section 2, researchers review the pertinent literature on FWB as well as its antecedents, and then discuss the development of these theories. Section 3 discusses the methodology of the study, followed by the findings and discussion in Section 4. Finally, in Section 5, researchers will conclude the study with a discussion of the implications, limitations, and recommendations.

## Literature review

### Financial Well-Being (FWB)

The term "financial well-being" refers to a level of financial security both now and in the future [44]. In accordance with Objective 3 of the Sustainable Development Goals, it is regarded as an effective instrument for combating poverty and fostering individual well-being. Academic fields as diverse as developmental psychology, consumer decision-making, economics, and financial advice and planning have all weighed in on the concept of FWB. Despite this, there is still no universally accepted metric or definition exists [45], and information on the origins of the concept as a whole is yet widely accessible [19, 46]. Predominantly, FWB pertains to an individual’s capacity to effectively manage their money, effectively navigate the challenges and opportunities presented by their financial situation, successfully accomplish their financial objectives, and possess the financial independence necessary to have a better judgement that enable them to feel great on their lives [47–49]. Taking this into consideration, [50] conceived that the total contentment of people’s with their financial status as FWB.

Despite the fact that FWB can be evaluated based on either objective or subjective criteria, [51], the choice of measurement is influenced by the overarching definition of the concept [52]. For instance, it was explained that income adequacy, net worth etc. are construct of FWB objective approach [53]. Within this context, indicating that it is more all-encompassing and may incorporate non-monetary features, [54–56], all employed a subjective approach, which is appropriate for defining and measuring the complex and individual phenomena of FWB.

In line with this belief, [19, 57] ascertained that although numerous factors affect FWB, people’ lack of financial knowledge or illiteracy may be a contributing cause to the financial difficulties that reduce FWB. On the other hand, [58] present the former argument, which prior studies on FWB focuses solely on these three categories of influencing factors: individual financial behaviour, interpersonal variables, and environmental factors. The effect of both competence and financial behaviour on Financial Decision Making, specifically FWB has not been well investigated in the existing literature. It is for this reason, [49] implied that poor financial behaviour is feasible as a ground cause of FWB. Some even consider that financial behaviour and FWB as a crucial factor associated with LOC [43]. While there are plenty of this trend (financial behaviour) immediately known in literature, wretched salary, inadequate financial knowledge, and huge liability were unveiled to be detrimental to heap range of financial issues that definitely weight down individuals and households FWB [59].

Taking that into consideration, growing research interest on young adults’ financial well-being saw that if individuals did not have stable financial situations when they were younger, this may have a negative consequence for their well-being, interpersonal and familial relationships, and their chances of successfully transitioning into the next phase of their lives [60]. In addition, [61] also ascertained that young individuals’ FWB effects not only their cognitive well-being but to include their academic achievement, work performance and level of life satisfaction as well. Alongside of the matter, socio-economic variables are also essential for FWB [47] as [21] also discovered that factors such as LOC, social class, gender, intelligence level, level of educational attainment, and status of employment all possess a critical responsibility in determining young adults’ FWB.

For instance, posit such like a sizeable number concerning younger individuals were dissatisfied with their current financial situations, had low FWB, struggled to maintain control over their monthly spending, were financially illiterate, and had difficulties managing their monthly expenditure [61]. Those individuals, on the other hand, who discussed and sought the counsel of others regarding sensible spending behaviour, abilities in saving, budgeting, and investing, had better financial outcomes. They were more optimistic about their financial situation and more likely to engage in healthy financial behaviours including saving, spending wisely, and keeping track of their expenditures, all of which boded well for their future wealth [19]. This presupposes that young adults who had positive financial habits modelled for them by their parents and other adults throughout their formative years will have better financial behaviour and FWB as adults, which they will have a greater chance of achieving their financial objectives [22]. As a matter of facts, People have a better chance of improving their own financial well-being when they have frequent interactions with their parents about money and witness how their parents make financial decisions.

### Low-income young adults’ financial behaviour and FWB

As previously mentioned, there were abundant numbers of research looking at FWB and the financial behaviours of young people or emerging adults. [18, 62], for instance used university or college students as their sample, while other studies surveyed working young adults between a particular age scope [8, 63]. Extant studies have their spotlight on youths and young adults since this population group has an significant responsibility, as the potential ‘captain’ who will steer the world into even more difficult circumstances, they deserve special attention.

Young adults confront a variety of obstacles throughout their lives and are in the process of acquiring a wide range of life skills as they make the transition from childhood to adulthood. The abilities to manage one’s career and finances are two of the most valuable life skills that can be acquired. Therefore, research that emphasize on the economic circumstances of young people particularly in the context of homes with low incomes are perceived to be worthwhile.

Existing studies on FWB and financial behaviour have predominantly focused their attention on the financial circumstances of vulnerable society, particularly low-income households [28, 29, 64]. However, it would appear that there are just a limited number of research that focus on a certain segment of the population that is vulnerable, specifically young adults who come from families with poor incomes. Thus, this paper will address this gap in the literature.

### Financial knowledge and FWB

The literature shows that the term financial knowledge has often been used interchangeably with financial literacy. Financial knowledge refers to the ability to comprehend, administer, and come to conclusions regarding matters pertaining to finance. Previous study also emphasized in their studies that financial knowledge refers to a person’s foundational familiarity with financial concepts [19]. It reflects a person’s financial history, from how they get their start to how they manage their money and give back to the community. In other words, it is the ability to evaluate all of one’s financial options objectively and then choose the best course of action based on those options [65]. Everyone needs basic financial literacy to make ends meet and avoid stress. People must to have a rudimentary understanding of basic financial concepts such as investing money, saving money, credit, rate of interest, inflation, and the pricing of consumer products. Financial knowledge can be classified as objective and subjective financial knowledge. An individual’s perceived level of knowledge or self-assurance on his/her ability to make sound financial choices is referred to as subjective financial knowledge. This is in contrast to an individual’s objective financial knowledge, which is related to the individual’s real financial understanding of financial concepts which is measured using objective measurements such as knowledge on interest rates, inflation rates, and time value of money. Subjective financial knowledge and objective financial knowledge are distinct in terms of their definition and measurement [66].

Other scholars have suggested that there are numerous conceptualizations of financial knowledge and financial literacy [67, 68]. While there appear to be differences in the conceptualization of financial knowledge, findings of previous research suggest that individuals that are more financially knowledgeable are more likely to have higher FWB [19, 57]. A person’s financial skill set and perspective can be greatly expanded via exposure to the world of finance. Financial literacy knowledge which relates to a range of financial abilities, including the ability to organize one’s own finances, understand basic ideas in finance, and deal with many types of financial institutions [69], may result in a boost in their FWB. Recent studies, however, argue that those who take the time to educate themselves about money are more likely to make wise financial decisions and exhibit positive FWB [70, 71]. In a similar vein, [19] described that enhancing a person’s financial knowledge is one of the best strategies to build and keep people’s FWB. This study postulate that young adults who come from low-income households will have a higher level of FWB if they possess higher levels of financial knowledge, as hypothesized below:

Ha1: The FWB of low-income young adults is positively influenced by financial knowledge

### FWB and Locus of Control (LOC)

The level of control that an individual has over their work and the beliefs that they hold towards their own achievement is referred to as LOC [72]. In a recent study by [71], one’s perception of one’s own agency in shaping one’s destiny was termed as locus of control (LOC). Both the internal and exterior aspects of LOC are important. Those who hold an internal LOC believe that they have some say in shaping and controlling the outcomes of future events in their lives, while those with an external LOC view such outcomes as predetermined by random, chaotic forces [30, 73]. Within this context, a high level of internal LOC (financial LOC) is associated with an individual’s conviction that his or her financial accomplishment or failure is directly attributable to his or her own efforts. Those with a high external LOC, in contrast, are inclined to blame their financial successes or failures on circumstances outside of their control, such as bias, the mistakes of others, circumstance, injustice or the external economic condition. It was observed that those who have a high internal LOC within themselves are more likely to be self-aware, to take pride in their accomplishments, are confident about their own talents and skills, and are able to resist covert attempts at persuasion [72].

While the concept of LOC has received some significant level of research interest in many parts of the world, despite its importance, there is a dearth of research into the financial LOC [22, 74]. According to the research that has been conducted in the past, LOC is one of the personality qualities that receives the most attention in the fields of personality and applied psychology [75]. Despite this, only in the past few years have social researchers begun to integrate LOC with financial conceptions in any significant way [22, 20]. As a direct consequence of this, a group of renowned researchers have investigated the link that exists between LOC and various financial notions such as FWB. Previous research has indicated that individuals’ financial and non-financial behaviours can be influenced by LOC [74, 76] particularly in terms of internal and external LOC exclusively. Incontrovertibly, LOC is given more weight in the study of individual monetary affairs than of business’s [30].

Those who experienced high levels of internal LOC are more likely to have higher FWB [22], whereas people with an external LOC seems to find themselves in financial difficulties [71, 77]. In a similar manner, [21] concurred with other researchers that LOC is an important factor influencing FWB. Other indications include parental social status, education level, and self-esteem. People who have a LOC that is internal to their organization are more likely to put in the effort necessary to accomplish what they set out to do. Therefore, an individual’s future wealth will be greater if they have faith in their own financial abilities, pay attention to the spending patterns of those around them, and have open conversations with their significant others about matters pertaining to money [22]. Thus, this study hypothesizes the following:

Ha2: The FWB of low-income young adults is positively influenced by internal LOC.

Ha3: The FWB of low-income young adults is negatively influenced by external LOC.

### Mediating role of financial behaviour

Common monetary behaviors include using cash, relying on credit, and saving. In a recent study by [20], financial behaviour was used to describe any action taken by a human being in relation to their relationship with money. This was implied previously by [30, 73] in their study which explained that financial behaviour may be broken down into four distinct categories: asset management, credit control, acquisition of capital, and general management. Previous research has shown that on-time bill payment, budgeting, debt management, expenditure strategy and pension planning have all been found to be indicators of healthy financial behaviour. Malaysian Financial Planning Council has raised concerns about the poor financial behaviour of Malaysians, to the extent of building up excessive debt, lack of budgeting and performing good financial behavior, which leads to higher financial distress and difficulties among Malaysians including young adults [78].

Due to the evidence suggesting that most young individuals are struggling with financial management and spending money to meet their daily financial commitments, and financial desires, scholars have examined the impact of young adults on their financial planning [20]. Result of the study that the majority of youngsters splurge their money on displaying their lifestyle and impressing others by purchasing items such as clothing, cosmetics, movie tickets, and meals at high end restaurants [79]. These poor financial behaviours and habits have a substantial influence on their personal lives and financial well-being.

Chong et al., On the contrary, contend that a firm knowledge of personal finance can help people make wise decisions such as obtaining personal and family health insurance [80, 81], spend on the needs and not wants [82], use credit card in a prudent manner [14] and paying bills on time [83]. Having said that, low financial literacy is a major contributor to the prevalence of financial difficulties, which can have serious consequences [57].

Previous research has shown that students with an external LOC have poor money management skills [79]. Thereafter, [84–86], also pointed out that one’s LOC has an impact on how they manage money. According to [70], higher levels of external LOC were associated with less responsible financial behaviour among college students. Contrarily, young adults who had a higher internal LOC and who believed they could influence the outcomes of their lives were the most responsible savers [30]. However, contradicted earlier research by arguing that locus of control does not influence individuals’ spending behaviors [87]. While most researchers agree that having a high level of internal LOC is linked to responsible financial behaviour, they are less certain about the effects of having a high level of external LOC. This research aims to fill in those gaps by testing the hypothesis that internal and external LOC would have an effect on the way young individuals handle their money.

To fill the gaps on the existing literature, this paper will examine the mediation of financial behaviour on financial knowledge internal and external, locus of control and financial well-being association amongst the young adults’ from low-income household. Drawing on previous researches, it is extremely important to improve one’s financial behaviour with LOC and financial knowledge [30]. Further, [57] also ascertained that a lack of knowledge about finances can lead to problems with individuals’ financial behaviours, such as strategies to manage their income, pension plan strategies, savings, and emergency plan, which can ultimately have an effect on their FWB [19]. Previous studies also have indicated that positive financial behaviour is associated with better FWB [80]. This paper serves as one of the efforts to test and validate the family resource management model, where the input and output effect is often mediated by intermediate processes [88]. Therefore, the purpose of this research is to assess how financial behaviour as the mediator between the independent and dependent variables. Therefore, we formulate the following hypotheses:

Ha4: Low-income young adults’ financial behaviour is positively influenced by financial knowledge

Ha5: Low-income young adults’ financial behavior is positively influenced by internal LOC

Ha6: Low-income young adults’ financial behavior is negatively influenced by external LOC

Ha7: Low-income young adults’ financial well-being is positively influenced by financial behaviour

Ha8: The association between financial knowledge and FWB of low-income young adults is mediated by financial behaviour

Ha9: The association between the internal LOC and low-income young adults’ FWB is mediated by financial behaviour

Ha10: The association between the external locus of control and low-income young adults’ FWB is mediated by financial behaviour

## Methods

### Sample

As part of a collaborative research project under the Malaysian Research University Network (MRUN), data were collected among low-income young-adult households in Malaysia. The sample for this research was selected using a multi-stage sampling procedure. In the first stage, households from four states (Selangor, Johor, Penang, Pahang) from five zones (Central, Northern, Southern, East Coast, and East Malaysia) of Peninsular Malaysia, including Sabah and Sarawak of East Malaysia were chosen using a random sampling technique. In the second stage, by using the National Household Sampling Frame (NHSF) and guided by the Department of Statistics Malaysia (DOSM), researchers managed to gather the respondent information relating to the young-adult’s socio-economic factors, savings, debt repayment and financial variables. 110 respondents represented each zone contributing a total of 550 respondents, which then only 520 data were accepted after cleaning the missing values and straight-lining errors.

The sample size for this study was determined using the criteria set forth by [89]. [90–91] both proposed an absolute minimum sample size of 100, whereas [92] considered sample sizes of 100 as inadequate, 200 sample size as acceptable, 300 as acceptable, and 500 as a maximum sample size to be the perfect sample required [93–95]. In order to account for any missing or incomplete questionnaires, the number of questionnaires distributed was increased by 20 per cent. According to the rule of thumb, the justification for 520 valid data used in the present study was considered sufficient and had an ideal sample size.

### Data collection

A cross-sectional study was performed to achieve the objective of the research. A pilot test was conducted among selected young-adult in order to ensure its precision and accuracy. Approximately 47 respondents completed the questionnaire, and based on their responses, the main instrument was revised to improve the terms and instructions sections, which the respondents needed help comprehending. Thus, the actual study’s results will be more reliable and accurate. In the first phase, a preliminary test was conducted to measure the content validity of the questions with the assistance of five academics and industry professionals. At this phase, the invited experts validated the contents of the instruments. In the second phase, 15 prospective respondents participated in in-depth interviews to recognize potential issue regarding to wording, clarity and format of the questionnaire. The predictable time required to complete answering the questionnaire was evaluated. Bedded by the response from the consultation, the researchers made some trivial modifications to the style and phrasing of the questionnaire. In the third phase, 47 participants from low-income household participated in a pilot study to signify the participants in the current research. According to the results, the final minor modifications were made and this study received approval from the Universiti Putra Malaysia Ethics Committee (UPMRMC) (Reference number: JKEUPM-2020-171). This study was conducted according to the ethical standards stipulated by the Declaration of Helsinki. All recruited respondents were required to sign the consent form before they proceeded to the questionnaire survey. Throughout the part of the data gathering procedure where actual information was gathered, enumerators made aware respondents before they filled out the survey. Respondents were briefed on the study’s goals and rationale, their role in it and the compensation they may expect to receive for participating voluntarily. Consensually, no monetary incentives were presented to participants. Time and financial constraints required the deployment of the sampling method and strategy. Furthermore, this method has no bearing on the reliability of statistical interpretations. Young adults in the range of 18 to 35 years old is the unit of analysis for this study. The reason of choosing the group is because they constitute a significant proportion of the population. Besides, statistics result also showed that majority of Malaysian young adult are declared bankrupt, and the numbers continue to rise over the time [96]. The results indicate, inferentially, that this particular population is particularly important for FWB.

It is worth mentioning again that the data in the present study were obtained using a set of questionnaire-based surveys. Thus, to address some possible bias that might occur, researchers used strategies to minimize the impact of a survey technique on the final results. First, the researchers took extra precautions in terms of sampling. According to [97], using a representative sample is critical for minimizing sampling bias. Thus, probability sampling methods such as simple random sampling can help ensure that the sample is representative of the studied population. In the present study, random sampling techniques were applied to address this issue. Second, researchers also used a set of questionnaire with validated measurement of the variables. All questions were very clear, concise, and free from ambiguity. This statement can be proven based on the values obtained from the validity and reliability tests. In addition, conducting a pilot test of the survey before the actual data collection phase can help identify and address any potential problems with the survey instruments. This step helped ensure that the questions were well understood by the respondents and that the survey measured what it was intended to measure [97]. Lastly, as argued by [98], the present study also did not provide any monetary incentives to the respondents to ensure that no incentives will influence the feedback from respondents, which can lead to inaccurate data.

### Instrument and measurement of variables

A self-administered survey was implemented in this research. There are five sections in the questionnaire consists of demographic and socio-economic profile, including questions on financial knowledge, financial behaviour, LOC and FWB. A significant challenge for researchers using regression analysis is ensuring their hypothesized model is free of endogeneity problems. Typically, this problem occurs when an independent variable correlates with the regression error in a regression model. In common usage, a variable possessing this characteristic is called an endogenous variable. In most cases, endogeneity occurs when an influential variable is omitted from the model, resulting in an omitted variable bias. The omitted variable is the most common cause of endogeneity [99–101]. It is possible to rectify the endogeneity problem by applying an instrumental variable regression (IV regression) [102].

### Financial knowledge

Ten (10) true and false questions were assessed to measure the level of respondents’ financial knowledge. The highest number with correct answer specifies high level of financial literacy. Ten questions were adapted from [103] which is a Malaysian-based scale development. Each item on the scale was related to one of five financial literacy domains: savings, investment, general financial knowledge, credit cards and products [104].

### Locus of Control (LOC)

LOC measurement was adopted from a study by [70]. The measurement comprises of eight items and four items for each internal and external LOC subscale. A Four-point Likert scale were used to measure the scale’s items. The answer ranges from Strongly Disagree (1) to Strongly Agree (4). A greater overall score on the internal subscale reflects a greater degree of internal LOC. In contrary, a lower overall score on the external subscale indicates a more effective external LOC.

### Financial behavior

The financial behaviour scale was developed for the Malaysian context conducted by the Malaysian Financial Planning Council [78]. Previous Malaysian financial study has also implemented and validated this measurement [104]. This question was evaluated using a four-point frequency scale (Never = 1 to Always = 4) and consist of eight positive and two negative items. The higher the level of financial behaviour, the higher the overall score represents.

### Financial Well-Being (FWB)

In this study, the FWB of adolescents was evaluated using eight items adapted from a questionnaire prepared for the Malaysian setting created by [105, 106] otherwise known as the Malaysian Financial Well-Being Scale (MFWBS) which then utilized by Mokhtar and Husniyah’s [107] study. This scale assessed respondents’ satisfaction with their overall financial status, their capability to cover everyday expenditures, their financial planning, their recent level of financial satisfaction and their financial sustainability. It comprises of eight positive items scored on a four- point Likert scale (Strongly Disagree = 1 to Strongly Agree = 4). Respondents that possess a greater degree of FWB have a higher total score.

### Conceptual model

**Fig 1:**
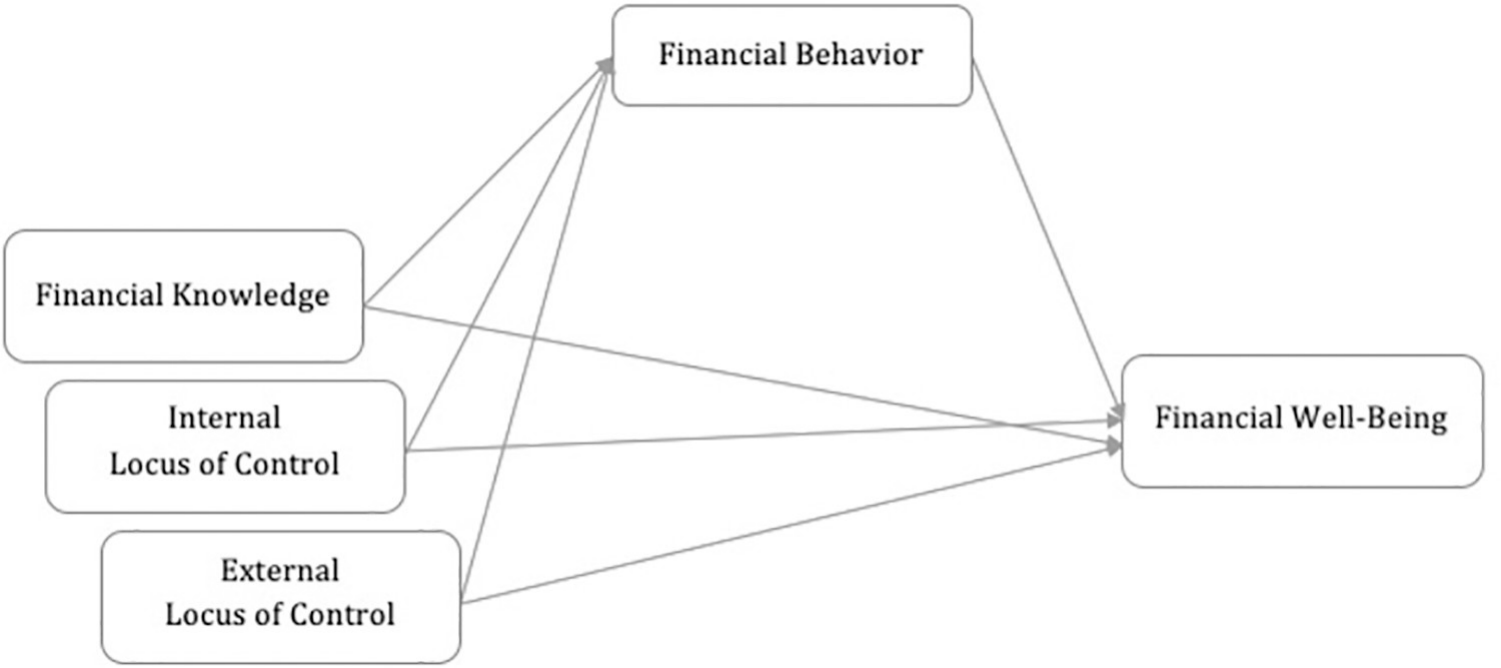
Hypothesized conceptual model.

In this study, a conceptual model was built to examine FWB among low-income young adults and its ability to characterize the interactions between four constructs which are internal and external LOC, financial behaviour, financial knowledge and FWB (see Fig 1). As illustrated in Fig 1, this study explored the direct and indirect impact of financial knowledge, internal and external LOC upon FWB. The relationship between the independent variables and the dependent variable is mediated by financial behaviour.

### Regression analysis

This study also estimate the determinant of financial well-being using the regression analysis as follows:

$$FWB_{i}=\alpha_{i}+\beta_{1}FB_{i}+\beta_{2}FL_{i}+\beta_{3}CE_{i}+\beta_{4}IC_{i}+\varepsilon_{i}$$ (1)

where FWB is financial well-being, FB is financial behaviour, FL is financial literacy, CE is the external LOC, IC is the internal LOC, and ε is the error term. The ordinary least square (OLS) method is used to estimate Eq (1). The idea that the error term is unrelated to the explanatory variable is a fundamental assumption in OLS. However, the correlation between the error term and the explanatory variable violates this key assumption of OLS, which results in inaccurate estimates or endogeneity problem. Endogeneity in an econometric model occurs when an explanatory (independent) variable is correlated with the error term (also known as the “disturbance term”) [108]. Without using the proper econometric methods to account for endogeneity, researchers risk finding "spurious correlations" between the explanatory variables and the dependent variable. Incorrect policy recommendations could result from failing to account for endogeneity, in addition to providing wrong coefficient estimates. The empirical research in the literature has identified three frequent endogeneity sources, namely simultaneity bias, omitted variable bias, and inaccuracies in variables.

This study investigates whether Eq (1) has omitted variable bias and simultaneity bias that causes the endogeneity issue. The omitted variable is the most common cause of endogeneity [99–101]. The simultaneity bias occurs when one (or more) explanatory variables are causally related and have simultaneous effects on each other [109]. For example, financial literacy (explanatory variable) and financial well-being (dependent variable) could all be determined simultaneously. Financial literacy and financial well-being variables can both occur at the same time, such as a person’s financial literacy depends on their current and future financial well-being. To test for endogeneity issue, the instrumental variables (IVs) method is used to correct for endogeneity brought on by omitted variable bias, reverse causality, simultaneous equations bias, or the presence of unmeasured confounding effects [102]. Although they do not serve as explanatory variables in the first-stage regression model, IVs are variables that are correlated with the endogenous explanatory variable(s) but are uncorrelated with the error term [110]. A popular method for IV estimation is the two stage least square (2SLS) approach. The use of IV-based estimation involves considerable theoretical and empirical explanation. For instance, researchers must conduct a few statistical tests such as Durbin and Wu-Hausman [111] to verify endogeneity. Endogenous factors must also be thoroughly evaluated and interpreted considering the available research and theory. Finding a valid instrument is crucial, both the choice and strength of a valid IV play an important role in the implementation of an IV. Researchers must ensure that the chosen instruments are relevant and exogenous before putting an IV estimation into the estimation. In this study, the education and income variables are used as IVs, where higher education and income are correlated with financial literacy.

To evaluate the relevance assumption, researchers can compare the R-square of the first-stage regression changes after additional instruments are included in the model. Technically, the subsequent changes in R-square should be significantly higher [100]. To determine the significance of an instrument, previous scholar proposed the first-stage F-statistics [112]. The specified instruments can be taken into account in the 2SLS, based on higher F statistics. On the other hand, weaker instruments are those with lower F statistics [113]. The Sargan [114] and Basmann [115] tests are employed in post estimation testing to assess the instrument’s over identification.

## Findings and discussions

### Data analysis

In this study, the data were analyzed using SPSS 26.0 software. Five (5) steps have been included on overall data analysis procedure. First, descriptive statistics have been performed by the researchers to categorize the univariate characteristics. Second, Cronbach’s alphas have been computed to indicate the consistency of each construct. Then, Pearson correlation test have been used to examine the association between all constructs. Subsequently, by employing a linear multiple regression analysis, the fitness of the FWB model were tested by researchers. Finally, three simple mediation models have been tested using the PROCESS macro for SPSS [116]. To establish the power of estimation, Bias-corrected bootstrap (n = 5000) confidence intervals, "the most trustworthy’ approach for significant testing were employed [117].

### Profile of respondents

This research was carried out among 18- to 35-year-old Malaysian young adults. Based on these totals, approximately 2.5% of the respondents were below 20 years old, 52.7% of them were in their 20’s (20 to 29 years), while 44.8% were in their early 30’s (30 to 35 years). There were approximately more than half (63.5%) are male and the rest are female (36.5%). Most of them are Malays (71.9%), then Sabah ethnicity (10.0%), other ethnicities (6.5%), Indians (6.0%), Sarawak ethnicity (2.7%), and the rest are Chinese (2.9%). In terms of marital status 57.3% of respondents are married, 38.3% are single, 3.5% are divorced, and 1.0% are separated from partners. For level of education, less than half of the respondents (46.5%) had high education (tertiary), and the other half had low education with primary and secondary school levels. Amongst the tertiary level of education respondents, 19.0% of respondents have diplomas, 16.9% had bachelor degrees, 2.3% had master degrees or doctor of philosophy, and another 8.3% had the Malaysian Higher School Certificate (STPM) or pre-university certificates.

### Reliability and normality tests

Reliability coefficients of the instruments used in this present study were identified based on the Cronbach’s alpha values, which derived from the reliability analysis done using the IBM SPSS. The reliability coefficients for the five constructs were ranged from Cronbach’s alpha 0.75 to 0.88, met the requirement that the Cronbach’s alpha must being greater than 0.7 [118]. Specifically, Cronbach’s alpha value for FWB is 0.88, Cronbach’s alpha values for constructs internal and external LOC are 0.78 and 0.82, respectively. The value of Cronbach’s alpha for financial behaviour is 0.81, while Cronbach’s alpha for financial knowledge is 0.75. In this study, the normality of the data was evaluated using the Skewness and Kurtosis statistical methods. Mentioned by several scholars, the appropriate range for skewness and kurtosis to confirm a normal univariate distribution is between -2 and +2 [119]. To determine if data are normally distributed, the + 2.58 criterion is appropriate when the sample size is more than 200 and the standard errors are small [120]. In accordance with the criteria mentioned above, the study measured absolute skewness values between -0.565 and 0.336 and kurtosis values between -0.462 and 0.673.

### Bivariate correlation analysis

Table 1 displays correlation coefficient between the variables in this research. In this particular study, every independent variable, with the exception of the external LOC, was found to have a significant positive correlation with FWB. The range values of all correlations are between 0.128 to 0.398. Specifically, Pearson correlation tests indicate that internal LOC (r = 0.291, p < 0.001), financial knowledge (r = 0.128, p < 0.01), and financial behaviour (r = 0.398, p < 0.001) results were positively effect with FWB, meanwhile external LOC was negatively and significantly correlated with FWB (r = -0.186, p < 0.001). The results also portrayed that internal LOC (r = 0.358, p < 0.001), and external LOC (r = -0.099, p < 0.05) and financial knowledge (r = 0.115, p < 0.01) have a significant correlation with financial behaviour. Hence, hypotheses Ha1, Ha2, Ha3, Ha4, Ha5, Ha6, and Ha7 were supported. Conversely, financial knowledge has a significant effect to both internal (r = 0.117, p < 0.01) and external (r = -0.205, p < 0.001) LOC in the positive and negative directions, respectively.

**Table 1:** Coefficient of correlations of variables.

	Financial knowledge	Internal LOC	External LOC	Financial Behavior
Financial Knowledge				
Internal LOC	0.117**			
External LOC	-0.205**	-0.311**		
Financial Behavior	0.115**	0.358**	-0.099*	
FWB	0.128**	0.291**	-0.186**	0.398**

Note: SD = standard deviation

*Significant at the 0.05 level (two-tailed)

According to Pearson correlation analysis, young adults of low-income background with high internal LOC and better financial knowledge appear to have a greater level of FWB. Besides, Furthermore, they appear to demonstrate a good financial behaviour. Young adults who have a greater external LOC, however, appear to have lower levels of FWB and financial behaviour. Furthermore, this research reveals that financial behaviour have a positive affect on FWB level of young adults from low-income background in Malaysian. These results clarify that a better financial behavior young adults appear to have greater FWB. According to the study done by [43, 121], these findings are similarly in line with previous studies, which showed financial knowledge has an impact on FWB and financial behaviour [15, 23, 122] of the individuals. Youngsters have a number of financial obligations and have only recently started their transition toward financial independence. Therefore, it is crucial to them to learn the basis of financial knowledge. It may affect their financial well-being as well as other parts of their lives. Hence, people should start learning about financial knowledge the young age. Financial knowledge can act as a form of early preparedness for individuals before entering the adult period.

The current research demonstrated that youngsters from low-income background with high internal LOC will experience greater FWB. On the other hand, those with a high external LOC will be, otherwise. People who had a strong internal locus of control oriented reported having a stronger sense of well-being while being in the middle of significant economic distress, as [49] also found in their study. The result is consistent with earlier research [22, 30, 123]. The vast majority of social science researchers have identified significant associations among external and internal factors of LOC with FWB. Youngsters who possess a higher LOC presume that they have a greater degree of control over their own destinies. As a consequence of this, they work to improve themselves in the hopes of leading more satisfying way of living. However, those who have a high external LOC believe that their lives are out of control. Thereafter, they surrender to their fates without even trying to upgrade their financial situations.

### Model fit

Using a regression model, we were able to identify significant components with FWB serving as the dependent variable. The model comprised financial knowledge, financial behavior, and internal and external LOC as predictive variables. FWB model fitness was analyzed using linear multiple regression analysis. The results indicated the R square = 0.197, which meant that 19.7% of the variance in FWB was described by the model. This result indicates that the independent variables accounted for 19.7% of the variance in FWB among young individuals who came from families with low incomes, with young adults’ financial behavior proving to be the most significant determinant. Furthermore, Cohen’s regression effect size, f square  =  0.245, for the overall model was found to exceed Cohen’s [124] convention for a medium effect f^2^ (= 0.150). Guidelines for interpretation of f square (f^2^) indicate that 0.020 is a small effect, 0.150 is a medium effect, and 0.350 is a large effect [124], indicating that the present effect size is medium to large. Based on the analysis conducted to test whether the data met the assumption of collinearity indicated that multicollinearity was also not a concern (Financial Knowledge, Tolerance = 0.956, VIF = 1.046; Internal LOC, Tolerance = 0.815, VIF = 1.227; External LOC, Tolerance = 0.903, VIF = 1.108; Financial Behavior, Tolerance = 0.866, VIF = 1.154). According to [89], to confirm that there is no evidence of multicollinearity, the value of variance inflation should be less than 10, and the tolerance value should be greater than 0.10.

Table 2 displays that the independent variables have a significant different to the dependent variable [F (4, 515) = 31.557, p<0.001]. Hence, the regression model is a good fit for the data. The same table also shows the prediction of financial behavior, financial knowledge, internal and external LOC. All variables in the model except financial knowledge were significantly influencing FWB. Based on the value of the standardized coefficients, the finding showed that financial behaviour is the most influential factor on FWB, followed by an internal LOC and an external LOC.

**Table 2:** Results of regression analysis for FWB Model.

Model	Unstandardized Coefficients		Standardized Coefficients	t	Sig.
	B	SE	Beta (ß)		
Constant	10.315	1.776		5.807	0.000
Financial Knowledge	0.170	0.131	0.053	1.300	0.194
Internal LOC	0.299	0.099	0.133	3.008	0.003
External LOC	-0.237	0.100	-0.100	-2.379	0.018
Financial Behavior	0.369	0.047	0.335	7.886	0.000

[F(4,515) = 31.672, p<0.001], R^2^ = 0.197, Adjusted R^2^ = 0.191

Note: Dependent variable (financial well-being), SE (standard error), p*<0.05, p**<0.01

### Mediation analysis

**Fig 2:**
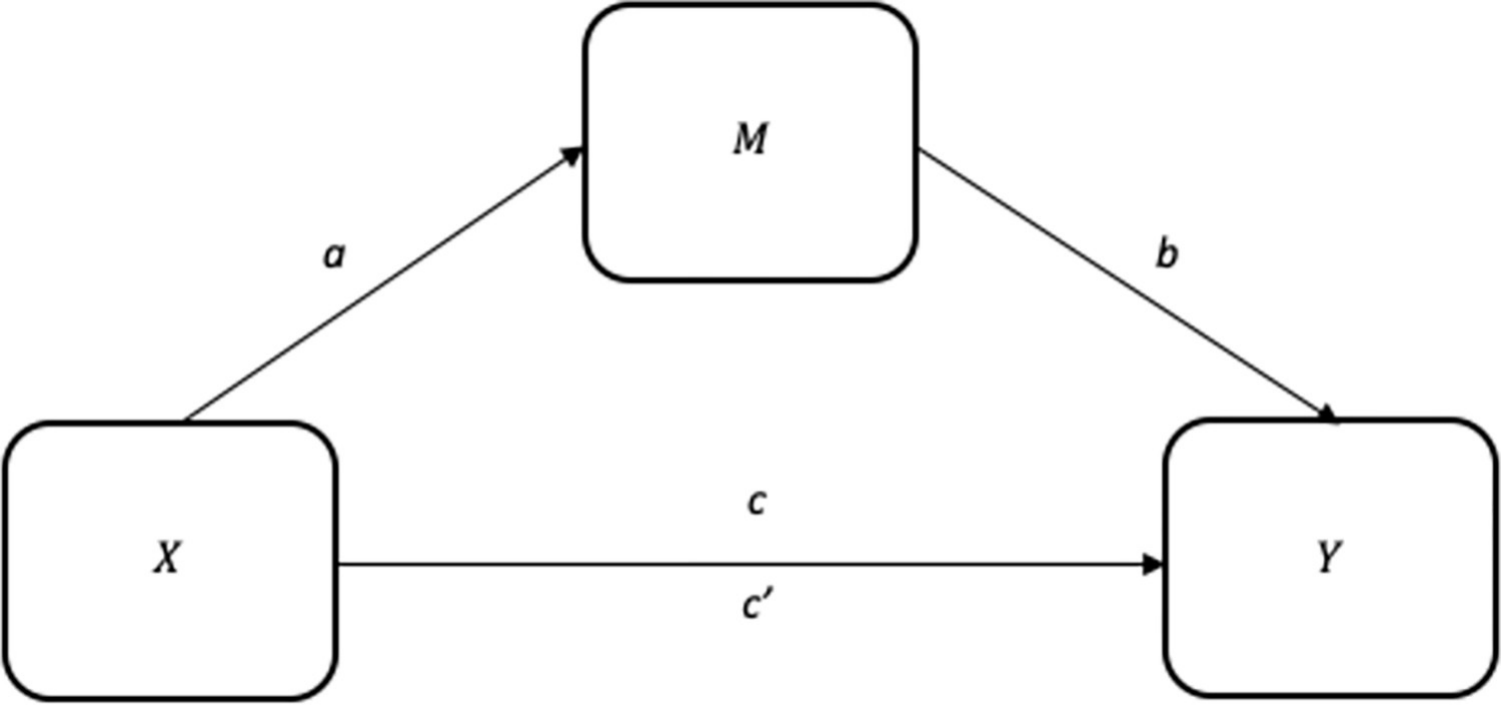
Simple mediation model.

In the present study, PROCESS macro method has been used to test the mediation hypotheses. PROCESS Macro is a bootstrapping statistical computer tool developed by Hayes [116] as an extension of IBM SPSS software. This method was utilized to explore how one or more mediating or moderating variables affect the relationship between the independent and dependent variables. The program calculates the direct (c), indirect (ab), and total effects (c’) of X on Y, as well as unstandardized and standardized regression coefficients, standard errors, and other statistics, including R^2^, t, and p values. Fig 2 shows the simple mediation model illustrating the relationship between X, M, and Y.

It’s feasible that the interaction between the independent and dependent variables is indirect or rather direct. Even though a third variable cannot have any effect on the correlation between X and Y, the direct effect is still present. On the other hand, an indirect impact takes place when the interaction between X and Y is formed (or mediated) by a third factor (s). The regression coefficients were represented in paths *a*, *b*, and *c*, between the X and M, M and Y, and X and Y correlation, respectively. The direct effect of X on Y indicated as the coefficient *c’*, whereas the indirect effect of X on Y indicated by the coefficients *a* and *b*. The total effect (*c’*) of X on Y is equal to *c* + a*b [125].

The results for the 3 simple mediation models in this research is shown in Table 3, while Table 4 shows the effect size for each path for all three models. A bootstrapping method was performed using SPSS Process Macro to examine whether financial behaviour mediated the relationships between financial knowledge (Model 1), internal LOC (Model 2), external LOC (Model 3), and FWB.

**Table 3:** Total, Direct, and indirect effects of financial knowledge, internal LOC, and external LOC on FWB (Bootstrap = 5000).

Effects	b (ß)	SE	t	p	Bootstrap Bias-Corrected 95% Confidence Interval		
					Lower Level (LLCI)		Upper Level (ULCI)
Model 1: Effect of Financial knowledge on FWB							
Total (c)	0.412 (0.128)	0.141	2.926	0.004	0.136		0.689
Direct (c’)	0.267 (0.083)	0.131	2.045	0.041	0.011		0.524
Indirect (ab)	0.145 (0.045)	0.017			0.041		0.260
Model 2: Effect of Internal LOC on FWB							
Total (c)	0.652 (0.291)	0.094	6.910	0.000	0.467		0.838
Direct (c’)	0.381 (0.170)	0.096	3.985	0.001	0.193		0.569
Indirect (ab)	0.272 (0.121)	0.058			0.168		0.393
Model 3: Effect of External LOC on FWB							
Total (c)	-0.438 (-0.186)	0.102	-4.310	0.000	-0.638		-0.239
Direct (c’)	-0.348 (-0.148)	0.094	-3.696	0.002	-0.534		-0.163
Indirect (ab)	-0.090 (-0.038)	0.044			-0.182	-0.006	

Note: Estimates in the parentheses were standardized values, LBCI = Lower Level Confindence Interval, ULCI = Upper Level Confidence Interval

**Table 4:** Coefficient model of financial behavior on the relationship between financial knowledge, internal LOC, external LOC and FWB.

Path (X→ M → Y)	Effects of X on M (a)	Effects of M on Y (b)	Indirect Effects (ab)	Direct Effects (c’)	Total Effects (c)	Bootstrap Bias-Corrected 95% Confidence Interval		
						Lower Level (LLCI)	Upper Level (ULCI)	p
FK → FB → FWB	0.338	0.428	0.145	0.267	0.412	0.011	0.524	0.041
ILoC → FB → FWB	0.730	0.372	0.271	0.381	0.652	0.193	0.569	0.001
ELoC → FB → FWB	-0.213	0.423	-0.090	-0.348	-0.438	-0.534	-0.163	0.000

Note: FK = financial knowledge, FB = financial behavior, ILoC = internal locus of control, ELoC = external locus of control, FWB = financial well-being, LLCI = lower level confidence interval, ULCI = upper level confidence interval

As for Model 1, regression analysis result showed that financial knowledge (independent variable) was a significantly influenced financial behavior (mediator: a = 0.338, t = 2.649, p < 0.01). Next, the results of the second regression analysis displayed that financial behaviour was a significant predictor of FWB (dependent variable: b = 0.428, t = 9.614, p ≤ 0.000) while controlling for financial knowledge. The outcomes of the indirect effect based on 5000 bootstrap samples displayed a significant indirect negative relationship between financial knowledge and FWB, mediated by financial behaviour [ab = 0.145, (bootstrap bias-corrected 95% CI: LLCI = -0.043 and ULCI = 0.265)]. Furthermore, there was a significant direct effect between financial knowledge and FWB (c’ = 0.267, t = 2.045, p > .05). With this significant indirect effect, the present study accepted Hypothesis 8 (Ha8). In line with previous study done by [116] who presented that the relationship between financial knowledge and FWB existed when there is mediation of financial behaviour variable. This result proved that the financial literacy of Malaysia’s low-income youngsters is indirectly correlated with their FWB via the relationship with financial behaviour.

Next, the result of the regression analysis for Model 2 also showed that the internal LOC (independent variable) was significantly correlated with financial behaviour (mediator: a = 0.730, t = 8.730, p ≤ 0.000). Then, while controlling for internal LOC, the result exhibited that financial behaviour was significantly correlated with FWB (dependent variable: b = 0.372, t = 7.985, p ≤ 0.000). The subtle effect result presented a significant correlation between internal LOC and FWB, with the mediation of financial behaviour [ab = 0.271, (bootstrap bias corrected 95% CI: LLCI = 0.164, ULCI = 0.400)]. Besides, the study also showed that the internal LOC and FWB direct effect was statistically significant (c’ = 0.381, t = 3.985, p ≤ 0.000). Therefore, the findings of this study led the researchers to the conclusion that financial behaviour acted as a mediator in the interaction among the internal LOC and FWB. The internal LOC among young individuals in Malaysia had an indirect correlation with FWB through financial behaviour. Thus, Hypothesis 9 (Ha9) is supported.

As for the last hypothesized model 3, the first regression analysis portrayed that independent variable (external LOC) was significantly correlated with financial behaviour (a = -0.213, t = -2.276, p < 0.05) in a negative direction. The second regression results also presented that financial behaviour (mediator) influenced FWB (dependent variable; b = 0.423, t = 9.588, p ≤ 0.000), while controlling for the external LOC. Based on 5000 bootstrap samples, the indirect effect result showed a significant and negative correlation between external LOC and FWB, mediated by financial behaviour (ab = -0.090, bootstrap bias-corrected 95% CI, LLCI = -0.182, and ULCI = -0.006). The results also revealed a significant direct effect between external LOC and financial behaviour (c’ = -0.348, t = -3.696, p ≤ 0.000). With this significant outcome, Hypothesis 10 (Ha10) was confirmed. The findings of this regression analysis demonstrated that financial behaviour is the mediating factor in the relationship between external LOC and FWB. Therefore, it was also elucidated that the young Malaysian adults’ external LOC is indirectly correlated with their FWB via its correlation with their financial behaviour.

According to the findings of this study, the majority of young adults with low incomes in Malaysia had difficulties attaining FWB due to their poor financial behavior and lack of financial knowledge. The vast majority of studies in the field of social sciences acknowledge that an increased level of financial literacy is one of the most important factors in improving financial behaviour, where eventually leads to an increase in FWB. The current finding is also consistent with a research amongst Malaysian working adults [13] including in previous researches [30, 31, 121, 126]. Furthermore, a Malaysian study done by [57] confirmed that financial resources and mismanagement of income led to more young adults in Malaysia filing for bankruptcy, which has a negative effect on not only the individuals involved but also their families and the country as a whole. This research reveals that in addition to financial knowledge, both internal and external LOC were indirectly connected with the FWB of Malaysian young adults with low incomes via their financial behaviour. This correlation was shown to have a considerable bearing on the topic. These findings are in line with the findings of other significant studies that has discovered a nexus between LOC, financial behaviour, and FWB [22, 43, 127].

Table 5 present the regression results of Eq (1). Model (1) is the OLS estimation, where the financial behavior is positive and statistically significant determinant of financial well-being at 1% level. Internal LOC and external LOC variables are significant determinants of financial well-being, with positive and negative signs, respectively. On the other hand, financial literacy indicates a negative sign but is insignificant determinant of financial well-being. The F-statistic is significant at 1% level, which reveals that the OLS model is valid and well-specified. Nevertheless, Eq (1) may have an endogeneity issue and the 2SLS is employed to address this problem. Model (2) reports the 2SLS result, the Durbin and Wu-Hausman tests [111] reveal that the null hypothesis of no endogeneity is failed to reject since both p-values are greater than 0.05 or 5% significant level. This finding demonstrates that there is no endogeneity issue and the OLS result is valid. Furthermore, the Sargan [114] and Basmann [115] tests of over identifying restrictions condition is valid, which confirms the instrument or model is correctly specified. In short, the model has no omitted variable bias or simultaneity bias since the financial literacy variable is exogenous in influencing financial well-being. The first stage result is also presented in Model (3) with two IVs, where the F-statistic and R-square are higher than the OLS. This finding suggests that the chosen instruments can be considered in the 2SLS regression.

**Table 5:** Results of OLS and 2SLS.

	Model (1)	Model (2)	Model (3)
Variables	OLS Dependent variable: FWB	Two stage least square (2SLS) Dependent variable: FWB	First Stage Dependent variable: FL
Financial Behavior	0.369***	0.392***	-0.046***
	(0.0467)	(0.0508)	(0.0155)
Internal LOC	0.312***	0.308***	-0.022
	(0.0982)	(0.1040)	(0.0321)
External LOC	-0.178**	-0.238***	0.0786***
	(0.0736)	(0.0901)	(0.0239)
Financial Literacy (FL)	-0.154	0.670	-
	(0.1340)	(0.5520)	
Education	-	-	0.701***
			(0.140)
Income	-	-	0.202**
			(0.095)
Constant	11.29***	9.296***	0.478
	(1.680)	(2.237)	(1.113)
Observations	520	495	495
R-squared	0.497	0.512	0.583
F-statistic	31.56***	36.11***	41.23***
Durbin chi-square test	-	2.5101 (p-value = 0.1131)	-
Wu-Hausman F-test	-	2.4923 (p-value = 0.1150)	-
Sargan chi-square test	-	1.2724 (p-value = 0.2593)	-
Basmann chi-square test	-	1.2602 (p-value = 0.2616)	-

Notes: Standard errors in parentheses.

***, ** and * denote significant at 1%,5% and 10% levels, respectively.

## Conclusion and implications

In this study, we build and evaluate a theoretical framework for FWB that incorporates individuals’ financial behaviours, financial literacy, and locus of control. This research is one of the earliest attempts to examine the interplay between financial literacy, internal and external locus of control, financial behaviour, and financial well-being amongst the Malaysia’s young adults with the background of low-income. The findings of this study also support the findings of prior studies that have found that increasing one’s financial literacy and one’s use of both internal and external LOC can lead to greater FWB among low-income young adults in Malaysia. Meanwhile, the findings also demonstrated that one’s financial behaviour is the most accurate determinant of one’s FWB, with both dimensions of LOC coming in second.

Among the contributions of this paper is to differentiate between the influence of internal and external LOC on the relationship with FWB, in the context of young adults from low-income households. The results show that financial behavior mediates the relationship between both dimensions of LOC towards FWB. Internal LOC is mediated by financial behaviour, with a positive sign, suggesting that one’s own control of financial matters will result in higher FWB through the conduct of positive financial behaviour. Meanwhile, external LOC is mediated by financial behaviour with a negative sign, suggesting that one who perceive that luck and external factors determine the outcomes in life, will have a weaker FWB through the conduct of poor financial behaviour. Both these results suggest that LOC is transmitted through financial behaviour in impacting FWB. Policy makers should provide the awareness to low- income young adults on financial matters, not only by providing financial education programs to the youths of low-income households, but also in terms of skills enhancement trainings. Assistance programs can be given to these young adults on starting their own business to take charge and control of their financial matters, rather than to succumb to the uncertainties of the economy and to simply allow these external factors influence the lives of these young adults. It is pertinent that these youths be given training on how to generate their own income such as entrepreneurial skills or skills enhancements such as in the field of technical and vocational educational training (TVET), which is currently being emphasized by the Malaysian government. These low-income young adults should be given priority in terms of government assistance programs in order to enhance their financial well-being and to take charge of their family’s welfare.

Despite not being a major finding, another interesting detail emerged from the investigation. Even though most of the young adults in this study have completed at least some higher education, they are still considered to be low-income because of Malaysia’s minimum wage regulation. Some even hold a master’s degree and doctors of philosophy. The relationship between high cost of living, low income, and FWB indeed a worthy discussion and should be explored by Malaysian policymakers and leaders. These matters requires to be the subject of forthcoming debate and to be further explore in Malaysia. In addition, taking into consideration the ongoing economic decline that has been going on for the past few years, as well as the younger generation’s high living standards, extreme debt, and organizing money matters that are having to confront the youth of today, the researchers evaluated the characteristic of young adult’s progression and seek to fully grasp by what means FWB be able to affiliated with behaviours and individual characteristics (i.e. LOC). Consequently, the researchers expanded an integrative model which examines the impact of FWB on the young adults by integrating behavioral and psychological perspectives.

A substantial proportion of young people in Malaysia experience unfavourable results in their financial life as a direct result of poor financial habits and an illiteracy about finances. The reasons for this may be traced back to a lack of education regarding finances and poor financial habits. As a result, the findings of this study suggest that acquiring financial knowledge via education scheme, be it formal nor informal, is essential for everyone in order to establish healthy financial habits or behaviours that lead to a better FWB. This is due to the current study that suggests attaining financial knowledge leads to a better FWB. Schools and other types of institutions ought to incorporate lessons on personal finance into their curricula. On the other hand, the process of establishing positive FWB is a delicate one that cannot be completed successfully solely by education on its own. It is influenced by a diverse range of aspects of young people’s socialization, and the young people themselves need to play an important part in addressing this issue. It is quite improbable that knowledge of finances, on its own, will have a substantial impact on either financial well-being or personal achievement in life.

Similarly, by setting a positive example, adults in a child’s growth can significantly contribute to the development of healthy financial behaviours and habits in that child. This is certainly relevant among younger generations. One should begin the process of enhancing their financial capability at a young age with the support of their closest systemic elements. Families and other associated local institutions have a responsibility to teach young people the importance of saving money and to discourage them from making rash, unexpected expenditures. They require direction in order to properly assess the many financial products, policies, or companies involved before making any decisions on their finances. Into the bargain, young adults should make it a priority to acquire crucial financial skills such as regular expense tracking, budgeting, saving and preparing for later stages of life, spending limitations and unexpected requirements, and primarily, stay within your financial means. It is also the government’s responsibility to make it easier for young people to consult with and receive advice from financial consultants and counsellors. If they make such initiative, they may be able to find credible guidance rather than misinformation on the Internet or from other questionable sources.

This study contributes to the current corpus of research by advocating in favour of ongoing public education initiatives that are directed toward teachers, parents, and students. The importance of information exchange and responsible financial behaviour should be emphasized in such programs. In fact, these efforts might be included into preschool and elementary school curricula to help kids learn to save and budget early on. Youngsters are better served by a curriculum that emphasizes real-world applications of financial management principles over abstract theories. So, youngsters can gain experience with money matters and improve their future financial well-being. The instillation of self-assurance in problem-solving by parents, educators, and peers are also vital, as it will help youth to make more informed economic decisions and better weather inevitable upheavals and setbacks.

### Limitations and recommendations

This study has two drawbacks that need to be addressed in subsequent research, despite the fact that it has few noteworthy discoveries and vital consequences for individuals, communities, and the government. Nevertheless, these findings and implications are essential. First, all of the Malaysian young adults who participated in our survey came from low-income backgrounds. Future research might compare the factors that influence FWB among different age groups, or look at how young adults from different socioeconomic backgrounds compare to one another. A comparative study would be beneficial in order to better understand the discrepancies in irresponsible financial behaviour that exist across the different age groups or household income categories.

Second, this research was carried out in the form of a cross-sectional study. Due to the use of cross-sectional data, this study was unable to investigate the causal linkages that exist between the variables in an appropriate manner. In order to investigate the possible causal relationships, longitudinal data are essential. Performing longitudinal research in countries with extensive secondary data on household finances may shed more light on FWB of young individuals over the long run. Additionally, insufficient secondary data to exist at the national level to properly assess the financial situations of Malaysian families is worth to note. Without a doubt, a causal model would aid in our comprehension of not only the evolution of FWB but also its beginnings and the socialization process, the individual and psychological aspects involved, and the different routes one may pursue into adulthood.

## Supporting information

S1 Data (XLSX)
