# Correction: An enigma of Malaysia’s low-income young adults: Mediation of financial behaviour on financial well-being and locus of control cohesion

**DOI:** 10.1371/journal.pone.0318382

**Published:** 2025-01-24

**Authors:** Mohamad Fazli Sabri, Rozita Wahab, Nurul Shahnaz Mahdzan, Amirah Shazana Magli, Husniyah Abd Rahim, Siti Shazwani Ahmad Suhaimi, Nur Shuhamin Nazuri

There are errors in the author affiliations. The correct affiliations are as follows:

Mohamad Fazli Sabri^1^, Rozita Wahab^3^, Nurul Shahnaz Mahdzan^2^, Amirah Shazana Magli^1^, Husniyah Abd Rahim^1^, Siti Shazwani Ahmad Suhaimi^1^, Nur Shuhamin Nazuri^1^

**1** Faculty of Human Ecology, Universiti Putra Malaysia, Serdang, Malaysia, **2** Department of Finance, Faculty of Business and Economics, University of Malaya, 50603 Kuala Lumpur, Malaysia, **3** Department of Educational Psychology and Counselling, Faculty of Education, University of Malaya, 50603 Kuala Lumpur, Malaysia
